# Systematic Transcriptome Wide Analysis of lncRNA-miRNA Interactions

**DOI:** 10.1371/journal.pone.0053823

**Published:** 2013-02-06

**Authors:** Saakshi Jalali, Deeksha Bhartiya, Mukesh Kumar Lalwani, Sridhar Sivasubbu, Vinod Scaria

**Affiliations:** 1 GN Ramachandran Knowledge Center for Genome Informatics, CSIR-Institute of Genomics and Integrative Biology (CSIR-IGIB), Delhi, India; 2 Genomics and Molecular Medicine, CSIR-Institute of Genomics and Integrative Biology (CSIR-IGIB), Delhi, India; University of Pennsylvania School of Medicine, United States of America

## Abstract

**Background:**

Long noncoding RNAs (lncRNAs) are a recently discovered class of non-protein coding RNAs, which have now increasingly been shown to be involved in a wide variety of biological processes as regulatory molecules. The functional role of many of the members of this class has been an enigma, except a few of them like Malat and HOTAIR. Little is known regarding the regulatory interactions between noncoding RNA classes. Recent reports have suggested that lncRNAs could potentially interact with other classes of non-coding RNAs including microRNAs (miRNAs) and modulate their regulatory role through interactions. We hypothesized that lncRNAs could participate as a layer of regulatory interactions with miRNAs. The availability of genome-scale datasets for Argonaute targets across human transcriptome has prompted us to reconstruct a genome-scale network of interactions between miRNAs and lncRNAs.

**Results:**

We used well characterized experimental Photoactivatable-Ribonucleoside-Enhanced Crosslinking and Immunoprecipitation (PAR-CLIP) datasets and the recent genome-wide annotations for lncRNAs in public domain to construct a comprehensive transcriptome-wide map of miRNA regulatory elements. Comparative analysis revealed that in addition to targeting protein-coding transcripts, miRNAs could also potentially target lncRNAs, thus participating in a novel layer of regulatory interactions between noncoding RNA classes. Furthermore, we have modeled one example of miRNA-lncRNA interaction using a zebrafish model. We have also found that the miRNA regulatory elements have a positional preference, clustering towards the mid regions and 3′ ends of the long noncoding transcripts. We also further reconstruct a genome-wide map of miRNA interactions with lncRNAs as well as messenger RNAs.

**Conclusions:**

This analysis suggests widespread regulatory interactions between noncoding RNAs classes and suggests a novel functional role for lncRNAs. We also present the first transcriptome scale study on miRNA-lncRNA interactions and the first report of a genome-scale reconstruction of a noncoding RNA regulatory interactome involving lncRNAs.

## Introduction

Recent advances in sequencing technologies have enabled genome-scale mapping of transcriptional potential feasible at single-nucleotide resolutions [1], [2]. A number of reports in the immediate past have hinted at the pervasive transcriptional potential of eukaryotic genomes [3], in addition to implicating a large number of novel genomic loci which were previously not known to encode any functional transcripts [4]. Though consistent evidence now suggests the existence of a large number of previously uncharacterized transcripts, a vast majority of the transcripts surprisingly do not code for a functional protein, and are grouped into a general class of noncoding RNAs (ncRNAs) [5]–[7]. The amenability of transcriptome mapping on a genome scale has enormously added to the number of ncRNAs, with members in the class presently outnumbering protein coding genes by a few folds. Recent efforts globally, including collaborative efforts such as the ENCODE [8] have been successful in characterizing the transcript structures and their expression profiles in a wide number of cell types. This has also been supplemented by the availability of computational tools to map and identify transcript isoforms and quantify expression profiles in different cell types and conditions [9].

The ncRNA class now encompasses a wide diversity of subclasses organized based on their size, structure, function and conservation. MicroRNAs (miRNAs) have been one of the recently discovered and well characterized classes of ncRNAs and are small regulatory RNA molecules processed from larger precursors through a highly coordinated pathway [10], [11]. Presently they are known to mediate post-transcriptional control of gene expression by binding to the 3′-untranslated regions of protein coding genes [12]–[14]. This is modulated through a ribonucleoprotein complex called the RNA induced silencing complex (RISC) [15]. Argonaute (Ago) proteins are the catalytic component of RISC complex. The small ncRNA bound to Ago guides it to sequence complementary target sites [16]. The regulatory role of miRNAs encompass a wide variety of biological processes including development, organogenesis [17], [18] and pathophysiology of a number of diseases including neuronal [19], cardiovascular [20] and infectious diseases [21]–[23], just to name a few. Another major class of recently discovered ncRNAs is the long noncoding RNAs (lncRNAs) [24], [25]. Unlike the miRNAs, lncRNAs are longer, and by definition >200 nucleotides in length and usually show less sequence conservation. lncRNAs presently also encompass the previously known classes of processed pseudogenes, antisense transcripts and the recently discovered regulatory large intergenic noncoding RNAs (lincRNAs) [26]. Though a small number of lncRNAs have been functionally characterized like HOTAIR [27], [28], Xist [29] and Malat [30], a large number of members in the class remain functionally uncharacterized. Presently well-characterized members of the class have been shown to be involved in regulatory roles in diverse processes like imprinting, X-inactivation and development. In addition, lncRNAs are also known to be associated with pathogenesis of a number of disease [31], recent reports from our lab also suggests potential of lncRNAs to be processed to smaller RNAs [32].

Though much of the focus in ncRNA research is directed towards understanding the regulation of protein-coding genes mediated by them, it has been suggested previously that ncRNAs could form a well-orchestrated regulatory interaction network [33]. There have been reports previously, which have suggested examples of such regulation, for example miRNA-miRNA interaction [34]. In addition, computational prediction of miRNA target sites suggest a widespread network of miRNA-lncRNA interaction [35]. Recently there have also been reports suggesting the possibility of a widespread interaction network involving competitive endogenous RNAs (ceRNAs) where ncRNAs could modulate regulatory RNA by binding and titrating them off their binding sites on protein coding messengers [36]. An example for this type of regulation is exemplified by HULC, an lncRNA expressed in hepatocellular carcinoma, which binds miR-372 and forms a regulatory interaction [37]. Similarly reports suggest that linc-MD1 could interact with miR-133 and miR-135 and promote muscle differentiation [38]. Similar examples have been described in plant systems and encompass target mimicry [39], [40]. The entire spectrum of ncRNA regulatory layer, especially the possibility of regulatory miRNAs-lncRNAs interactions remains unexplored.

We employed datasets in public domain for lncRNA annotations recently made available from the ENCODE project along with genome-wide cross-linking with immunoprecipitation (CLIP-Seq) datasets for four Ago proteins [41]. We hypothesized that miRNAs and lncRNAs could form an interacting regulatory layer exemplifying the ceRNA hypothesis where multiple targets with multiple binding affinities compete for the regulatory RNAs and the members could modulate the regulation of each other by modulation of their relative concentrations (Figure S1). We show that a subset of the annotated lncRNAs could indeed harbor miRNA recognition elements and be a part of the miRNA interaction network, thus working as a critical regulatory layer of interaction. In addition, we have experimentally validated the interaction of *7sl* lncRNA with miR-125b in a zebrafish model, thus adding confidence to our hypothesis. Furthermore, we have also integrated miRNA-target information to suggest a network of interactions between lncRNAs, miRNAs and protein-coding transcripts. To our knowledge this is one of the first transcriptome scale study on miRNA-lncRNA interactions and the first report of a genome-scale reconstruction of a noncoding RNA regulatory interactome involving lncRNAs.

## Results and Discussion

### Identification of High-confidence microRNA Recognition Elements Mapping to Long Noncoding RNAs

The PAR-CLIP reads for HEK293 cells were downloaded from the NCBI Short Read Archive (SRA IDs: SRX020783, SRX020784, SRX020785, SRX020786), which amounted to a total of 19,301,715 CLIP-seq reads for Ago 1-4. The reads were further mapped to hg19 version of the human genome using a quality-aware reference mapping algorithm: Mapping and Assembly with qualities (MAQ). Mapping was performed with stringent criteria and allowed no mismatches. The Agos significantly overlap in their biological function. Ago1 has been shown to be involved in RNA-mediated post-transcriptional gene silencing, Ago2 shows slicer activity, Ago3 and Ago4 are required for RNA-mediated gene silencing (RNAi). Since there have been no significant differences reported between the mappings of reads for each of the Ago datasets, and since biologically their functions overlap, we merged the datasets as previously used for the analysis [42]. A total of 619,208 hits were obtained for the Ago datasets. The number of reads mapping in each of the individual datasets is available in Table 1. To weed out any potential noisy loci, we applied stringent criteria that all loci should be supported by at least 5 overlapping reads. The mappings were parsed to identify high-quality miRNA recognition elements using custom scripts. We defined the recognition element as a stretch of contiguous positions in the genome, which matched the 5× read mapping criteria. A total of 15,983 such consensus high confidence miRNA recognition element clusters were identified. These mapped to 113 lncRNA exons and 4,367 mRNA exons from the GENCODE annotation datasets [43]. We further also analyzed the relative contributions of each of the Ago datasets for each of the high-confidence miRNA recognition elements.

**Table 1:** The number of reads mapping in each of the individual datasets.

Protein Name	Read Length	Total No. of Reads	Reads mapped
Ago1	32	4155911	104075
Ago2	32	4588647	197799
Ago3	32	4122692	146122
Ago4	32	6434465	171212

### Identification of the Cognate microRNAs for the microRNA Recognition Elements

**Figure 1:**
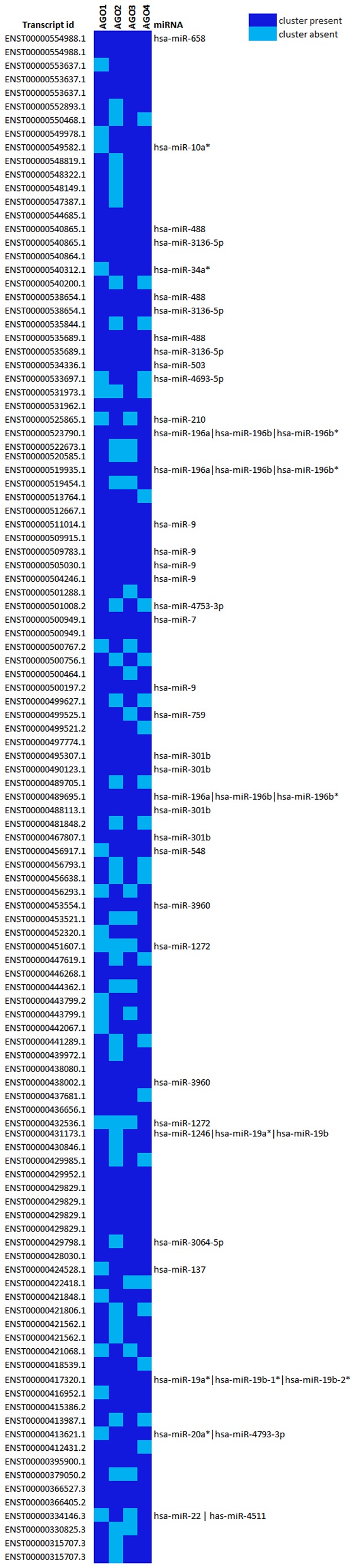
Depicting lncRNA transcripts targeted by miRNAs. The figure shows the lncRNA transcripts targeted by miRNAs along with the distribution of Ago types in the targeting complex. The presence and absence of specific Ago type in the RISC complex is represented by the difference in the colors in the heatmap. The targeting miRNA has also been mentioned.

Ago proteins are a component of the RNA induced silencing complex and are intricate components modulating the function of the complex. CLIP-seq allows one to identify the miRNA recognition elements in the transcriptome. To understand the biological interactions between miRNAs and their cognate target transcripts, it is imperative to identify the miRNAs corresponding to each of the miRNA recognition elements. We used miRanda [44] a popular computational algorithm to identify miRNAs binding to cognate miRNA recognition elements. Analysis revealed 51 miRNA-lncRNA interactions with 29 miRNAs targeting 25 lncRNA transcripts (Figure 1) (Table 2). We also predicted miRNA targets for all GENCODE annotated transcripts predicted to be bound with Agos. Out of the total 4,367 mRNA clusters, 416 protein coding transcripts were found to be targeted by 1,253 miRNA (Figure S2). The paucity in the number of known human miRNAs from the present analysis could potentially be an effect of the paucity in the biological understanding of the repertoire of smallRNA species in humans and their biological mechanisms. Apart from miRNAs, other smallRNA species also has potential to be associated with Ago proteins [45]. We hope future in-depth understanding of smallRNA species, including their biogenesis and mechanism of action could potentially throw light into the biological relevance of the remaining regulatory motifs.

**Table 2:** Contribution of each Ago class in targeting lncRNA.

Argonaute	Number of Ago binding sites present in lncRNA	Number of lncRNA transcripts	miRNAs
Ago1	91	80	hsa-miR-1246, hsa-miR-196a, hsa-miR-196b, hsa-miR-196b*, hsa-miR-19a*, hsa-miR-19b-1*, hsa-miR-19b-2*, hsa-miR-301b, hsa-miR-3064-5p, hsa-miR-3136-5p, hsa-miR-3960, hsa-miR-4753-3p, hsa-miR-488, hsa-miR-503, hsa-miR-658, hsa-miR-7, hsa-miR-759, hsa-miR-9
Ago2	75	65	has-miR-4511, hsa-miR-10a*, hsa-miR-137, hsa-miR-196a, hsa-miR-196b, hsa-miR-196b*, hsa-miR-19a*, hsa-miR-19b-1*, hsa-miR-19b-2*, hsa-miR-20a*, hsa-miR-210, hsa-miR-22, hsa-miR-301b, hsa-miR-3136-5p, hsa-miR-34a*, hsa-miR-3960, hsa-miR-4693-5p, hsa-miR-4793-3p, hsa-miR-488, hsa-miR-503, hsa-miR-548, hsa-miR-658, hsa-miR-7, hsa-miR-759, hsa-miR-9
Ago3	94	82	hsa-miR-10a*, hsa-miR-1246, hsa-miR-137, hsa-miR-196a, hsa-miR-196b, hsa-miR-196b*, hsa-miR-19a*, hsa-miR-19b-1*, hsa-miR-19b-2*, hsa-miR-20a*, hsa-miR-301b, hsa-miR-3064-5p, hsa-miR-3136-5p, hsa-miR-34a*, hsa-miR-3960, hsa-miR-4693-5p, hsa-miR-4753-3p, hsa-miR-4793-3p, hsa-miR-488, hsa-miR-503, hsa-miR-548, hsa-miR-658, hsa-miR-7, hsa-miR-9
Ago4	90	78	has-miR-4511, hsa-miR-10a*, hsa-miR-1246, hsa-miR-1272, hsa-miR-137, hsa-miR-196a, hsa-miR-196b, hsa-miR-196b*, hsa-miR-19a*, hsa-miR-19b-1*, hsa-miR-19b-2*, hsa-miR-20a*, hsa-miR-210, hsa-miR-22, hsa-miR-301b, hsa-miR-3064-5p, hsa-miR-3136-5p, hsa-miR-34a*, hsa-miR-3960, hsa-miR-4793-3p, hsa-miR-488, hsa-miR-503, hsa-miR-548, hsa-miR-658, hsa-miR-7, hsa-miR-759, hsa-miR-9

### *7sl* RNA lncRNA is Down Regulated by mir-125b in Zebrafish

**Figure 2:**
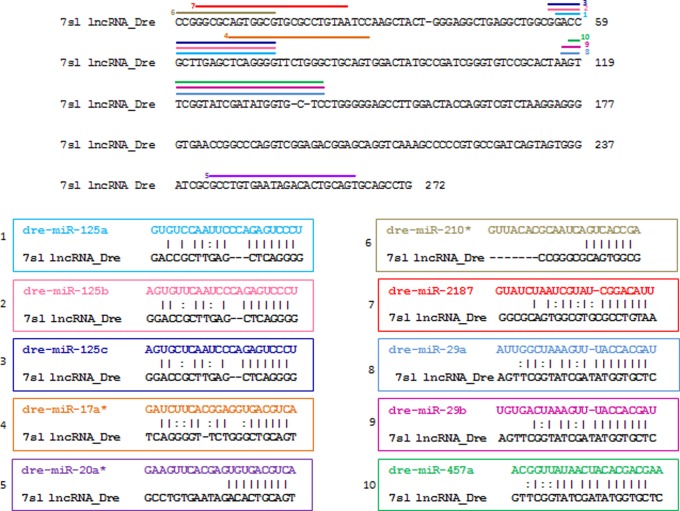
Binding alignments of microRNAs targeting predicted zebrafish lncRNA. The figure shows predicted binding alignment of miR-125a, miR-125b, miR-125c, miR-17a*, miR-20*, miR-210*, miR-29a, miR-29b and miR457a with predicted zebrafish lncRNA.

We have attempted to model one example of miRNA-lncRNA interaction in a zebrafish model. Initially, we performed BLAT analysis of human lncRNA against zebrafish genome for identification of putative lncRNA in zebrafish as described in method section. The BLAT analysis generated a single hit on zebrafish chromosome no. 3 (chr3:30399858-30400130), which had 91.64% similarity with human 7sl lncRNA. The miRanda software identified potential binding sites of miR-125a, miR-125b, miR-125c, miR-17a*, miR-20a*, miR-210*, miR-2187, miR-29a, miR-29b and miR-457a in the predicted zebrafish *7sl* lncRNA (Figure 2). MiR-125b is known to express ubiquitously during zebrafish embryogenesis [46]. In addition, miR-125b is known to targets *p53* mRNA to regulate embryonic development and stress response in zebrafish [46]. Therefore, we were intrigued to study the interaction of miR-125b with the predicted *7sl* lncRNA in developing zebrafish embryos.

**Figure 3:**
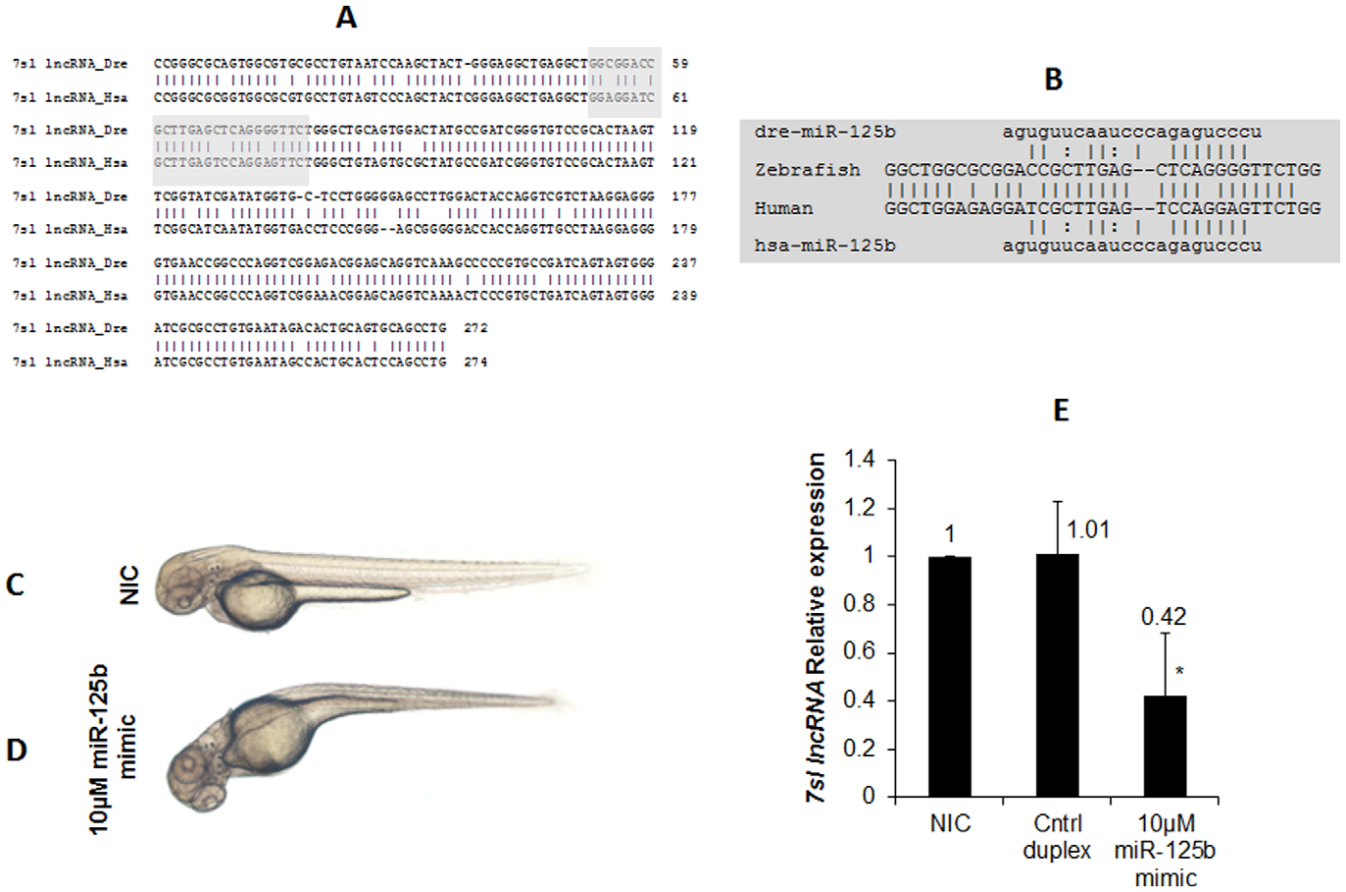
*7sl* RNA lncRNA is down regulated by mir-125b in Zebrafish. **A**: Sequence alignment of human *7sl lncRNA* with the predicted zebrafish lncRNA. The predicted miR-125b target region is highlighted in gray color. **B**: Predicted binding alignment of miR-125b with human *7sl lncRNA* and the predicted zebrafish *lncRNA*. **C** and **D**: Representative image of non-injected (NIC) and anterior posterior axis curvature defect in zebrafish embryos at 2 dpf. C) Non-injected control embryo (NIC) and D) miR-125b injected embryo. **E**: Relative quantification assay in 2dpf zebrafish embryos for the predicted zebrafish *7sl* lncRNA. Non-injected control (NIC), control mimic (miR-144) and miR-125b mimic. Data collected from 3 independent experiments is represented as mean fold change. ± SD. Asterisk (*) indicates p value of 0.01 as determined by 2-tailed t-test.

Prior to examining the interaction of miR-125b with the predicted *7sl* lncRNA in developing zebrafish embryos, we investigated the expression of the predicted zebrafish *7sl* lncRNA during zebrafish embryonic development using reverse transcriptase PCR technique. The predicted zebrafish *7sl* lncRNA was expressed in 24 and 48 hpf (hour post fertilization) zebrafish embryos (data not shown). The interaction of miR-125b and *7sl* lncRNA was investigated by ectopic overexpression of miR-125b mimic in zebrafish embryos [47]. Ectopic overexpression of miR-125b (10 µM) resulted in anterior-posterior axis curvature defects in approximately 80% of the injected zebrafish embryos at 2 dpf (n = 225/283) (Figure 3C and 3D). A sequence unrelated miRNA (miR-144) was used as control. Ectopic overexpression of control miR-144 (10 µM) did not result in any observable phenotype in zebrafish embryos at 2 dpf. Further expression of the predicted *7sl* lncRNA was assayed in miR-125b injected embryos using quantitative real-time PCR. MiR-125b injected embryos displayed reduction in expression of the predicted *7sl* lncRNA by approximately 1.72 fold (0.42±0.26 SD) compared to non-injected embryos (NIC) (Figure 3E). However, the expression levels of the predicted *7sl* lncRNA were not significantly affected in case of control injected miRNA (Figure 3E). In summary, we show that over expression of miR-125b resulted a corresponding decrease in the expression levels of the predicted *7sl* lncRNA in zebrafish embryos.

### Enrichment of microRNA Regulatory Elements in Long Noncoding RNA Ends

Analysis of the positional preference of miRNA regulatory elements in lncRNAs revealed a positional preference of the Ago binding sites in mid regions and at the 3` ends of the lncRNAs (Figure S3). Such preferential positioning of the miRNA regulatory elements have been described previously for protein-coding transcripts [48], with clustering in the 3′-untranslated regions of protein-coding genes. The biological significance of positional clustering in the ends of lncRNAs is uncertain at present though suggesting a possible pattern in organization of regulatory elements across transcripts.

### Long Noncoding RNAs as a Part of the microRNA Interaction Network

**Figure 4:**
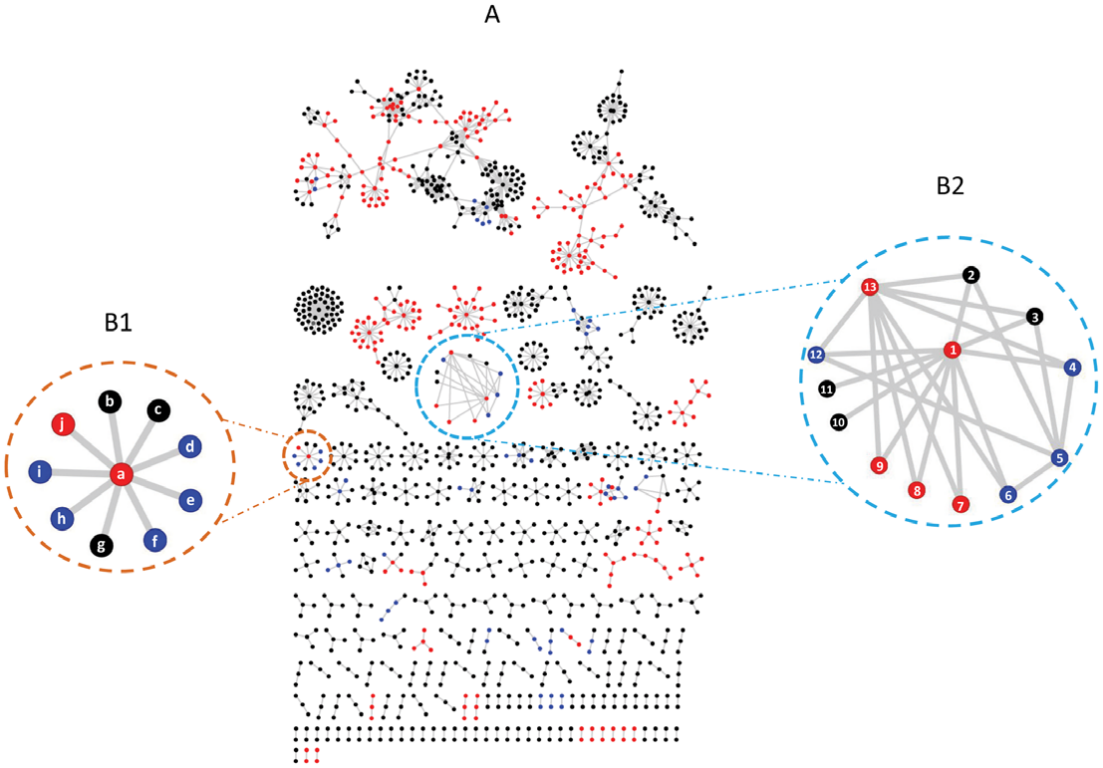
A: Complete interaction network of lncRNA, miRNA and mRNA. The interaction network wherein the experimental miRNA-mRNA interactions are represented in red nodes, the predicted miRNA-lncRNA interactions are represented in blue nodes and total miRNA-mRNA interactions represented as black nodes. **B1:** An interesting example from the network highlighted in orange showing interactions between of network highlighting **miRNA:** (a) hsa-mir-9; **lncRNA:** (d) ENST00000500197.2, (e) ENST00000509783.1, (f) ENST00000511014.1, (h) ENST00000505030.1, (i) ENST00000504246.1; **mRNA:** (b) ENST00000384838.1, (c) ENST00000262095.2, (g) ENST00000491143.1, (j) ENST00000226574 **B2:** Another interesting example from the network highlighted in blue showing interactions between **miRNA**: (1) hsa-miR-196a, (5) hsa-miR-196b*, (13)hsa- miR-196b; **lncRNA**: (4) ENST00000523790.1; (6) ENST00000489695.1, (12) ENST00000519935.1; **mRNA**: (2) ENST00000354032.4, (3) ENST00000384852.1, (7) ENST00000313173, (8) ENST0000024215, (9) ENST00000040584, (10) ENST00000304786.7, (11) ENST00000366839.4.

The present analysis suggests lncRNAs also harbor potential miRNA regulatory elements and could participate in the miRNA regulatory network. We reconstituted a comprehensive genome-wide network of RNA mediated interactions putting together the interactions from genome-wide analyses and validated miRNA-mRNA interactions. The network brings to light hitherto unknown complexity of RNA regulatory interactions and how lncRNAs could play regulatory roles in the miRNA mediated interactions with mRNAs (Figure 4). The reconstituted interaction network has 314 miRNAs targeting 35 lncRNAs and 946 mRNAs. The interaction network consists of several small and large clusters. To exemplify how lncRNAs could participate in the miRNA-mRNA interaction network, we have considered an example of hsa-mir-196a, which is experimentally known to target ENST0000040584, ENST00000242159, ENST00000313173 mRNA transcripts, encoded by the HOX cluster genes HOXC8, HOXA7 and HOXD8 [49], We noticed that the hsa-mir-196a could also target lncRNAs ENST00000519935, ENST00000523790, and ENST00000489695 (Figure 4B2). The mir-196 miRNAs are known to directly cleave HOX mRNAs and modulates development of axial patterning [49], [50]. The human miRNA hsa-mir-196a has been previously shown to be associated with the pathogenesis of cancers including colorectal cancer cells and has been shown to induce a pro-oncogenic behavior in human cancer cells [51]. Thus lncRNAs could potentially modulate the pathogenesis of the disease by modulating the key partner, mir-196a. We hope with the increasing understanding of the key roles in different diseases processes, a network approach would allow to prioritize the functional studies of lncRNAs in disease processes.

## Materials and Methods

### Ethics Statement

Zebrafish experiments were performed in strict accordance with the recommendations and guidelines laid down by the CSIR-Institute of Genomics and Integrative Biology, India. The protocol was approved by the Institutional Animal Ethics Committee (IAEC) of the CSIR Institute of Genomics and Integrative Biology, India (Proposal No 45a). All necessary efforts were made to minimize animal suffering.

### Zebrafish Husbandry

Zebrafish used in this study were housed at the CSIR-Institute of Genomics and Integrative Biology following standard husbandry practices [52]. Zebrafish embryos were obtained by pair-wise mating of adult fish.

### PAR-CLIP Datasets

We used transcriptome wide interaction data for miRNA-Ago available from PAR-CLIP studies [53]. The dataset provides the RNA-binding proteins (miRNA-Ago) target sites identified from the human embryonic kidney (HEK) 293 cell lines stable expressing Ago1-4. The read sequences (SRA IDs: SRX020783, SRX020784, SRX020785, SRX020786) of this study were fetched from Sequence Read Archive (SRA) from NCBI and were assembled using MAQ [42] reference assembly software to the hg19 version of the human genome from UCSC Genome Browser [54]. To identify only high-confidence miRNA recognition elements, we used cutoff of 5× and all loci with equal to or more than 5X coverage were filtered for the further analysis.

### Long Noncoding RNA Datasets

The manually annotated lncRNAs from GENCODE version 9 (May 2011 freeze, GRCh37) (http://www.gencodegenes.org/) [43] were used for the analysis. The dataset contains both Ensembl and Havanna annotations and the transcripts fall into six biotypes, viz, processed transcripts, antisense, lincRNA, ncRNA host, noncoding and retained intron. The dataset had a total of 18878 long noncoding transcripts encompassing by 11004 genes. The former dataset of high-confidence miRNA recognition elements were mapped on the lncRNA exon positions using custom scripts.

### MicroRNA Sequence Datasets

The dataset of mature miRNA sequences was derived from miRBase17 [55], [56] for all annotated Human miRNAs. This dataset comprised of 1731 sequences. The dataset of validated miRNA targets were derived from miRecords [57].

### MicroRNA Target Predictions

To identify the subset of miRNAs for the corresponding high-confidence Ago sites, we used a computational approach using a miRNA target prediction algorithm miRanda [44]. MiRanda uses dynamic programming and incorporates the free energy of the RNA duplex and miRNA binding rules to predict potential miRNA target sites. We used an empirical alignment score of 160 and minimum free energy of −20 kcal/mol. Similarly miRNA targets were predicted for all those messenger RNAs which harbored high confidence miRNA recognition elements.

### Positional Preference of microRNA Regulatory Elements in Long Noncoding RNAs

The positional preference of miRNAs across the lncRNA length was computed using in house scripts. We divided the entire length of the lncRNA into non-overlapping bins of 10 percent each. This was done to accommodate the diversity in lengths of the lncRNA. The frequencies of mapping miRNA regulatory elements were plotted for each of the bins.

### MicroRNA-interaction Network

**Figure 5:**
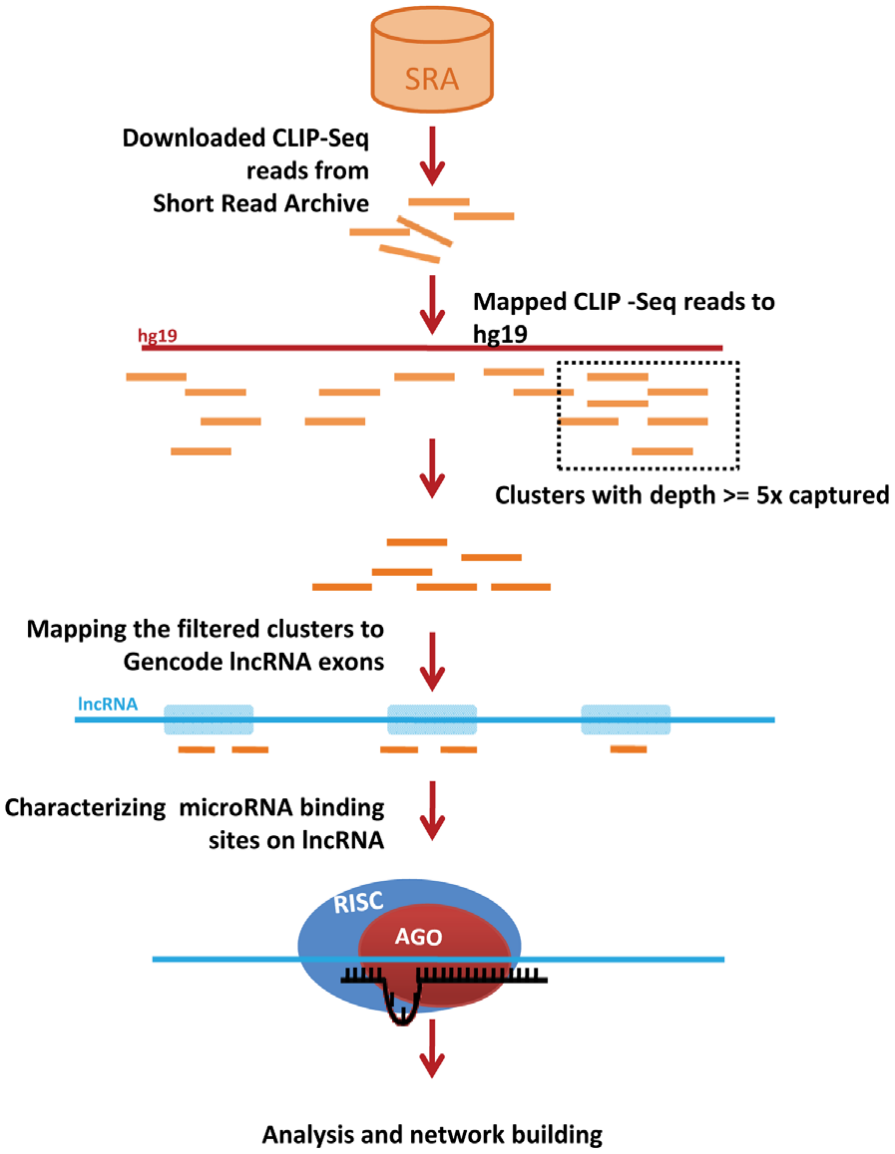
Workflow for the analysis of potential microRNA binding sites on lncRNAs and re-construction of the regulatory interactome.

Both the interactions, viz, miRNA-lncRNA and miRNA-mRNA were compared and an unweight network displaying miRNA-lncRNA and miRNA-mRNA interactions were created using Cytoscape 2.8.2 [58]. The targets were then searched on miRecords to check whether any of the high-confidence miRNA recognition elements overlap with validated target sites. The miRNA-mRNA interactions were also taken from literature. The entire workflow of the computational analysis is summarized in Figure 5.

### Prediction of lncRNA in Zebrafish

Human long noncoding RNA (lncRNAs) sequences were downloaded from lncRNAdb database [59]. These sequences were further aligned to zebrafish genome (Zv9) using UCSC BLAT tool to retrieve sequences with ≥90% identity score. The alignment percentage was calculated by dividing the aligned length by query (lncRNA) length. The zebrafish genomic regions with identical human lncRNA sequences were further analyzed with ensembl genome browser for expressed sequence tag (EST). The resultant sequences were considered as putative lncRNA in zebrafish. These sequences were further analyzed for miRNA binding site as mentioned in previous section (microRNA target predictions).

### Microinjections into Zebrafish Embryos

MiRNA injections were performed following a published protocols [47], [60]. Glass capillary (World Precision) micropipettes were pulled using Sutter Instrument (USA) and clipped appropriately for microinjection to deliver 2–3 nl solution into one cell stage zebrafish embryos. For overexpression of miR-125b mimic (Dharmacon), two to three nanoliter (nl) containing 10 µM miRNA mimic was injected at one cell stage zebrafish embryos. The concentration and dose of miRNA that was microinjected in zebrafish embryos were based on the previous published studies [47], [61], [62].

### Imaging

The injected and non-injected control embryos were observed and imaged in white light with an upright Zeiss Axioscope 40 microscope (Carl Zeiss, Germany) using 2.5X magnification and 0.075 numerical aperture. Images were processed with Zeiss AxioVision 4.6 and Adobe Photoshop CS software. Identical modifications and adjustment were applied to all the images in the same experiment.

### Quantitative Real-Time PCR (QRT-PCR)

RNA derived from miRNA injected and control embryos were obtained using Qiagen miRNeasy Mini Kit (cat no. 217004) according to manufacturer described protocol. One microgram of RNA was reverse transcribed into cDNA using Superscript II (Invitrogen, USA), and diluted by 1∶5 for RT-PCR assay. The assay was performed using Sybr Green mix (Roche, Germany). The oligonucleotide primers used for RT-PCR assay of predicted zebrafish *7sl* lncRNA was p1942F (5′-GTAATCCAAGCTACTGGGAGGC-3′) and p1943R (5′-AGGCGCGATCCCACTACTGATC-3′). The fold change were calculated by ΔΔCT method [63]. U6 used as an internal control using primers U6F (5′-ACTAAAATTGGAACGATACAGAGA-3′) and U6R (5′-AAAGATGGAACGCTTCACG-3′).

### Conclusions

The present analysis illustrates the potential integration of genome-wide experimental datasets to provide insights into novel biological regulatory interactions in the cell. Interactions for miRNAs with lncRNAs have been suggested to play an important role in modulating regulation mediated through miRNAs. This analysis also provides a genome-scale report and reconstruction of miRNA interaction with mRNAs as well as lncRNAs. We also show how such a network approach could potentially suggest the lncRNAs for functional validation. In addition we have also experimentally validated a miRNA-lncRNA interaction using a zebrafish model, which suggest that miRNA-lncRNA interaction networks are biologically active (Figure 3). We have earlier suggested distinct patterns of epigenetic marks across lncRNAs with potential significance in the transcriptional regulation of lncRNAs [64]. This present analysis adds to the understanding of yet another regulatory layer at the post transcriptional level modulating lncRNA expression and thereby the function.

The study is not without caveats. The first being limitation in the diversity of CLIP-seq datasets available in public domain. The datasets used in the analysis are limited to HEK293 cells. The second major caveat is that majority of miRNA recognition elements could not be tracked back to their cognate miRNAs. With the advent of faster and cheaper sequencing technologies [65], [66] we hope newer genome-wide CLIP-seq datasets would emerge. This would potentially provide a better picture of the depth of interactions and a perspective on the spatio-temporal organization of these networks. We hope the advancements in technology together with further insights into the biological function of small RNA classes including discovery of other members of this class would provide a better insight into the regulatory ramifications. This could also potentially pave the way to identify cognate RNAs for the regulatory elements discovered through genome-wide screens. We also hope the availability of technology to identify targets of RNA binding proteins would open up newer avenues for understanding the functions and targets of many of the RNA binding proteins. This would also provide for the much needed base to understand biological processes and regulatory interactions of ncRNAs with coding transcripts as well as between them in addition to deciphering how these proteins modulate these through binding to their respective partners.

## Supporting Information

Figure S1 **Schematic of the proposed hypothesis.**
(TIF)

Figure S2 **List of all transcripts with the Ago binding sites.**
(DOC)

Figure S3 **Distribution of Ago binding sites across the length of lncRNAs divided in bins of 10 percent. A)** The panel shows the number of Ago sites in each bin. **B)** The panel shows the individual Ago binding sites in lncRNAs transcripts.
(TIF)
