# Supplementary figures and images for: Systematic Transcriptome Wide Analysis of lncRNA-miRNA Interactions
Source: PLoS One. 2013 Feb 6;8(2):e53823. doi: 10.1371/journal.pone.0053823 (PMC3566149; doi:10.1371/journal.pone.0053823)

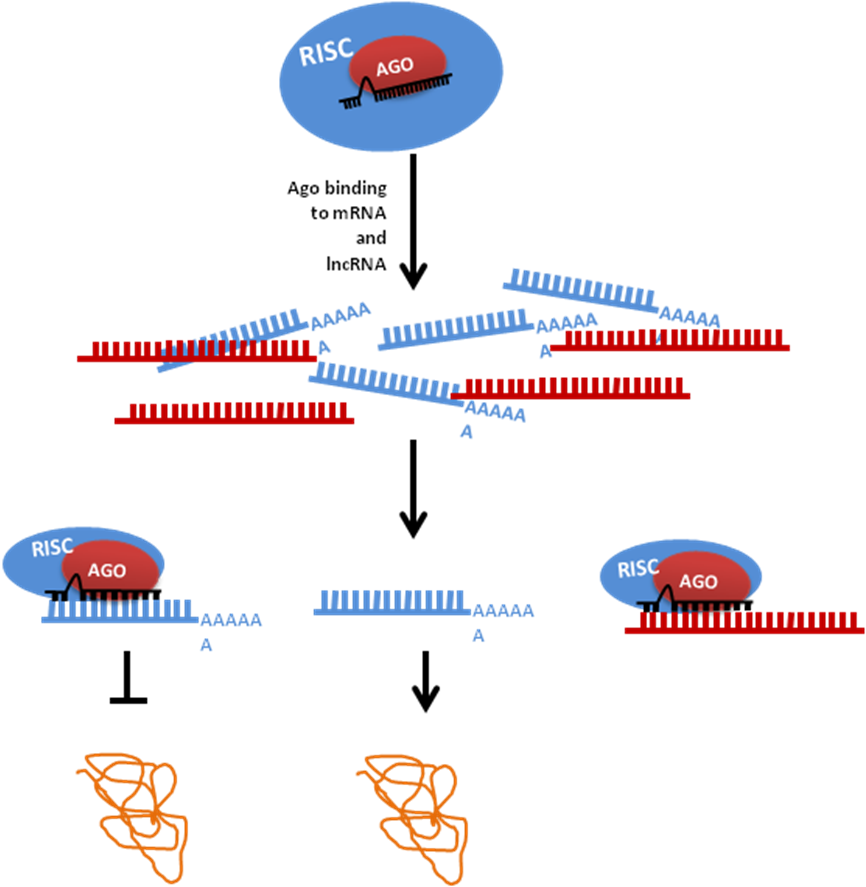

Supplement: Figure S1 — Schematic of the proposed hypothesis. (TIF) [file pone.0053823.s001.tif]

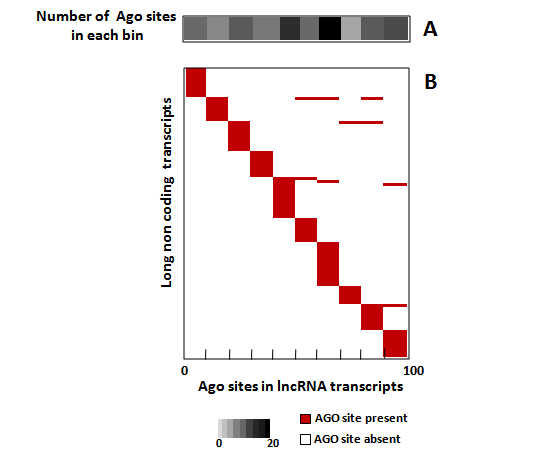

Supplement: Figure S3 — Distribution of Ago binding sites across the length of lncRNAs divided in bins of 10 percent. A) The panel shows the number of Ago sites in each bin. B) The panel shows the individual Ago binding sites in lncRNAs transcripts. (TIF) [file pone.0053823.s003.tif]
